# Physiological Costs of Repetitive Courtship Displays in Cockroaches Handicap Locomotor Performance

**DOI:** 10.1371/journal.pone.0143664

**Published:** 2015-11-25

**Authors:** Sophie L. Mowles, Natalie M. Jepson

**Affiliations:** 1 Department of Life Sciences, Anglia Ruskin University, Cambridge, United Kingdom; 2 School of Life Sciences, The University of Nottingham, Nottingham, United Kingdom; University of Natural Resources and Life Sciences, Vienna, AUSTRIA

## Abstract

Courtship displays are typically thought to have evolved via female choice, whereby females select mates based on the characteristics of a display that is expected to honestly reflect some aspect of the male’s quality. Honesty is typically enforced by mechanistic costs and constraints that limit the level at which a display can be performed. It is becoming increasingly apparent that these costs may be energetic costs involved in the production of dynamic, often repetitive displays. A female attending to such a display may thus be assessing the physical fitness of a male as an index of his quality. Such assessment would provide information on his current physical quality as well as his ability to carry out other demanding activities, qualities with which a choosy female should want to provision her offspring. In the current study we use courtship interactions in the Cuban burrowing cockroach, *Byrsotria fumigata* to directly test whether courtship is associated with a signaler’s performance capacity. Males that had produced courtship displays achieved significantly lower speeds and distances in locomotor trials than non-courting control males. We also found that females mated more readily with males that produced a more vigorous display. Thus, males of this species have developed a strategy where they produce a demanding courtship display, while females choose males based on their ability to produce this display. Courtship displays in many taxa often involve dynamic repetitive actions and as such, signals of stamina in courtship may be more widespread than previously thought.

## Introduction

During courtship interactions, males typically produce displays that are assumed to advertise some aspect of their quality to the female. The function of static displays of color and size are well understood in communicating overall health and the ability of the signaler to sequester resources [1, 2]. The evolution of exaggerated static traits via sexual selection and female preferences for trait elaboration has also received great attention historically [3, 4]. Thus, we now know a great deal about the function and evolution of ornaments in the context of courtship and mate choice. However, while many courtship displays involve the presentation of such ‘static’ traits, many also rely on the performance of dynamic displays involving the production of acoustic, motion, electric, seismic or other types of signals, all of which may be produced in bouts of repeated signaling. Dynamic repeated displays have been much studied under the framework of contest theory, where several models explaining the function of agonistic signal repetition have been developed [5–8]. The function of dynamic repeated courtship displays, however, is still relatively poorly understood and has only recently started to receive attention [9], despite potentially serving to communicate similar information to repetitive displays in contests: information about signaler quality [7, 9].

Dynamic repetitive courtship displays fall into two broad functional categories: either repeated displays allow information to be transmitted again (signal validation), or the process of repetition itself provides a demonstration of the signaler’s ability to withstand signaling costs (demonstration of cost capacities) [7, 9]. There may be both extrinsic and intrinsic costs of signal production during courtship. Extrinsic costs are related to the ability of the individual to devote time to producing a conspicuous display [10], which could otherwise be spent foraging, whilst at the same time exposing themselves to an increasing chance of being detected by predators [11, 12] or parasites [13, 14].

While extrinsic costs are often considered to be the circumstantial costs or ‘by-products’ of a courtship display [15], intrinsic costs are those that are incurred as a direct biological result of producing the signal. In the long term, intrinsic costs may relate to allocation during development and would thus be considered indices of quality (e.g. body or ornament size), but in the short timescale of a courtship interaction, intrinsic costs would relate to an individual’s energy metabolism. Thus, by performing multiple repetitions of a dynamic signal, an animal can demonstrate its energetic cost capacity (stamina). In many cases, it appears that ornamental traits and dynamic repetitive displays interact, where the investment in a large and often cumbersome ornament may act as a performance handicap to the display in which it is used. For example, the elongated tails of male swordtail fish, *Xiphophorus* sp., result in increased energetic expenditure from drag during swimming [16], yet are incorporated into a repetitive courtship display that females find attractive [17]. A female attending to such a display may thus be assessing the performance capacity of a male as an index of his quality. Females are likely to prefer males with higher energetic cost thresholds as this may indicate: i) that the male is an effective forager and has built up enough energy reserves to expend in display [11], ii) that the male has ‘good genes’ [1] for physical fitness and thus has the ability to perform other demanding activities such as dispersing, evading predators and defeating rivals; qualities with which a choosy female should want to provision her offspring [18] and iii) that the male is in good condition (i.e. not diseased or parasitised [19, 20]. Thus, signals communicating performance capacities are likely to be of huge importance to female mate choice. Indeed, it appears that females do attend to the rate of energetically costly courtship displays, such as the rate of drumming performed by wolf spiders, *Hygrolycosa rubrofasciata* [21, 22], the rate of claw-waving produced by fiddler crabs, *Uca* spp. [23, 24] and the rate of stridulation performed by the crickets, *Gryllus bimaculatus* [19, 25, 26] and *Teleogryllus commodus* [27, 28].

An alternative function of signal repetition is for signal validation, where repetition provides the opportunity for information to be transmitted again. Signal validation assumes that there is error in the production or transmission of the signal, due to environmental noise for example [29]. Repetition reduces this error by allowing the female further opportunities to assess male quality by repeated exposure to static characteristics such as ornaments or body size [5, 7, 9]. As the signal validation process is one of confirmation, female assessment is not predicted to be related to the rate of display [7, 9].

When we analyze potential signals of stamina from a mechanistic perspective, we must typically consider the physiology of energy metabolism, either by recording the expenditure of energy reserves or the accumulation of metabolic by-products associated with the production of a display [23, 26, 30–32]. However, many physiological and morphological factors are likely to interact in influencing an individual’s physical fitness. Studies of performance capacities or ‘whole-organism performance’ [33–36] can resolve this by measuring the amount of activity that an individual is capable of undertaking. By stimulating an animal to undergo strenuous activity on motorized treadmills [37, 38] or around circular racetracks [39–41], either up to its maximal rate of performance or maximal endurance, investigators can replicate the animal’s capacity to perform demanding ecologically relevant tasks such as evading predators (e.g. sprint capacity) or engaging in lengthy pursuits (e.g. tests of stamina). Investigations using performance techniques have revealed the links between repeated displays and stamina in the contexts of aggression [34, 38, 42], and predator deterrence [39]. However, such techniques have only recently been applied to courtship [43] and have not been explicitly used to investigate the function of dynamic repetitive courtship displays.

**Fig 1:**
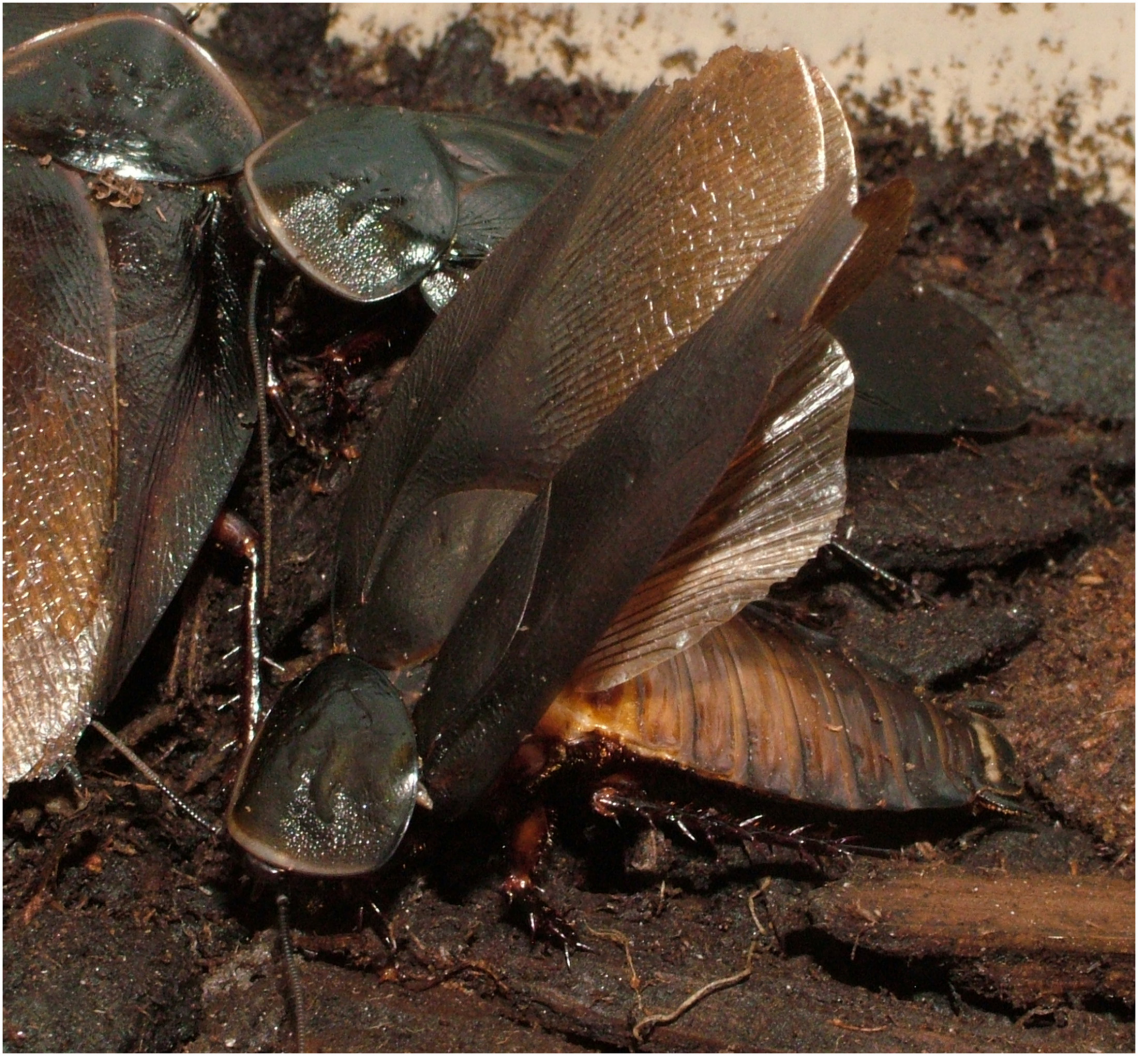
A courting male *Byrsotria fumigata* performing a full wing raise.

The Cuban burrowing cockroach, *Byrsotria fumigata* is tractable for use in performance capacity trials and also performs a dynamic repeated courtship display. In order to court females, male *B*. *fumigata* perform a preliminary display termed ‘wing-pumping’, which involves repeatedly raising the wings to an angle of approximately 10 to 20 degrees off the abdomen [44]. If the female appears receptive, the male commences full courtship behavior where he engages in the ‘full wing-raising display’ [44]. Here, the male proceeds to face away from the female and performs repeated exaggerated wing-raises, where he elevates his wings fully to a nearly vertical position (Fig 1). If courtship is successful, the female then mounts and mates with the male. As female *B*. *fumigata* mount the males, there are no apparent forms of sexual coercion as exhibited by species such as sagebrush crickets, *Cyphoderris* spp. [45]. Thus, it is possible to study female mate choice in this system in the absence of coercive mating. The function of the repetitive wing-raising display is currently unknown. The process may facilitate the diffusion of sex pheromone from the male’s tergal glands [46], or females may be responding to repeated presentations of the wings to gain accurate information on a morphological trait advertising the size of the male in accordance with signal validation. Alternatively, the vigour of the wing-raising display may advertise the capacity of the male to bear signaling costs.

The aim of this study is to apply whole-organism performance techniques to the repetitive courtship display performed by *B*. *fumigata*. In doing so, we directly test whether the repetitive courtship display is associated with the performance capacity of the male. We also carry out mate choice trials in order to investigate what aspects of the male's morphology and courtship display predict female mate-choice decisions.

## Material and Methods

### Study organisms

Cuban burrowing cockroaches were purchased from an entomological supplier (virginiacheeseman.co.uk) as nymphs and allowed to mature in a temperature-controlled laboratory maintained at 28°C on a 12:12 hour light/dark reversed cycle. Cockroaches were sexed once they began to exhibit sexually dimorphic subgenital plates, whereupon they were split between all-male and all-female tanks. This process ensured that only virgin cockroaches were used in this experiment. All cockroaches received a diet of *ad libitum* rabbit food and dog biscuits supplemented by fresh carrots.

### Prolonged courtship trials

In order to investigate whether courtship is fatiguing, we carried out trials where a male was introduced to a tethered stimulus female. Preventing the female from mating with the male allowed us to obtain courtship observations over the same period for each male before investigating the male’s current locomotory capacity in subsequent performance trials.

Behavioral trials were carried out at 28°C under white fluorescent laboratory lighting. Only animals without visible damage were used in the behavioral trials. Males were assigned to either the courtship group (*N* = 25 males) or the non-displaying control group (*N* = 25 males). Each male was photographed alongside a scale bar to accurately measure body and wing length by image analysis using ImageJ software (http://rsbweb.nih.gov/ij). It was ensured that the control group and display group did not significantly differ in size (mean body lengths ± s.e.: control, 3.944 ± 0.036 cm; display, 3.976 ± 0.052 cm (*t* = -0.551, *df* = 48, *P* = 0.5845), mean wing lengths ± s.e.: control, 3.176 ± 0.037 cm; display, 3.194 ± 0.037 cm (*t* = -0.344, *df* = 48, *P* = 0.7323)). Each male was individually placed into one side of a plastic container (185 mm L x 125 mm W x 75 mm D) divided in half by an opaque partition and left to acclimate overnight (16 h). Each male was provided with a piece of moist cotton wool and crumpled paper for shelter.

Twenty minutes before the observation period, the shelter and cotton wool were removed and a tethered female *B*. *fumigata* was introduced into the vacant half of the arena. Each female was tethered to the floor of the box using fishing line (approx. 10cm long), which was attached to the pronotum using duct tape. This allowed the female the ability to move, but not to rise up enough to mount the male [26]. Once the dividing partition was removed, the male was allowed to court the female for 360 s. The time period of 360 s was chosen as this is four times the typical display duration. Such a protracted courtship should allow the resolution of whether this constitutes an energetically costly display. Control encounters were staged in the same way, except that a female was not included in the arena. The empty half of the box had a small amount of tape and fishing line attached to the floor in order to control for any odors from the materials influencing male behavior. Each interaction was recorded from above using a Canon LEGRIA HF R26 High Definition Camcorder for the accurate quantification of courtship behavior (see ‘Analysis of courtship behavior’ section below). Following each interaction, the females were untethered without damage and placed individually into plastic containers with food, water and shelter until the mate choice trials the following day.

### Performance capacity trials

Immediately after the courtship or control trial, each male was transferred to a circular raceway (circumference 72.22 cm) and stimulated to run for five minutes by performing a series of light taps on its dorsum using a cardboard probe. The probe was wide enough to fill the width of the raceway, thus stimulating the cockroach to run continuously forward. Once in motion, the cockroach was pursued by the probe, which was always kept at one body length behind the running cockroach [41] and forced to run at its fastest possible pace, without being pushed by the probe. The cockroach only received taps by the probe when it stopped moving as intermittent locomotion has been shown to increase work capacity in invertebrates [47]. As cockroaches have high endurance (e.g. 90+ minutes of running for *Gromphadorhina portentosa* (pers obs and [48])), the total distance completed in five minutes was recorded for each male, rather than time until exhaustion [34, 41]. This yielded two measures of whole-body performance capacity: the overall distance attained in 5 minutes (overall rate of performance) and the maximum speed attained (m/s for fastest lap; a measure of maximum exertion achieved). The five minutes of running did, however, result in a drop in speed to a stable level, and the cockroaches covered approximately 38 m in this time.

### Mate-choice trials

Following the performance capacity trials, twenty males, ten of which had previously courted while ten of which had previously been used as controls) were placed back into their containers, provided with water, cover and a small amount of food and again left to acclimate overnight. We chose to use a mixture of males that had previously courted and those that had not in order to also test whether previous courtship effort depleted the subsequent ability to court the following day. Following the 16 h acclimation period, the female that each male had previously displayed to was introduced into the other side of his container, behind the opaque partition. The previously encountered female was used in order to standardize a male’s courtship effort across trials. This controlled for differences in male motivation due to different female characteristics (e.g. size, age or gravidness). Previous control males received a novel female. This time, each female was untethered. Following a ten minute acclimation period, the partition was removed and the male was allowed to court the female. No motivational difference was anticipated between the males encountering novel or familiar females as all males and females used in the trials were virgin stock and should be motivated to mate with the individual that they encounter. Indeed, motivation upon encountering a female that may have previously appeared as unreceptive (as she did not mate with the male) in the prolonged courtship trials was not an issue (see Results). Mate choice encounters were allowed to proceed for 15 minutes and either ended naturally due to female choice, or were terminated once 15 minutes had elapsed. In these latter cases the males were deemed unsuccessful at attracting the female.

### Analysis of courtship behavior

The footage recorded from each courtship and mate-choice encounter was later scored for behaviors using JWatcher behavioral observation software [49]. Courtship activities were measured from the point at which the barrier was removed and the cockroaches were allowed to interact. The male was scored for the vigour of the ‘full wing-raising display’ he performed. In the courtship trials, we recorded the frequency (the number of full wing-raises per trial) and duration of full wing-raising performed by the male. In the mate-choice trials, we recorded the frequency and duration of full wing-raising performed by the male and whether the male was successful in mating with the female. As the mating trials were of non-uniform length, we recorded courtship vigour as the proportion of time spent performing full wing raises until the conclusion of the trial. Longer trials would naturally enable the males to perform more wing raises or a greater overall duration of wing raises. Thus, using a proportion of time allows for the quantification of courtship effort in variable trial length.

### Statistical methods

Data were analyzed using StatView 5.0 (SAS, Cary, NC, U.S.A.) and R version 3.1.0 was used to build the GLMs.

#### Analysis of prolonged courtship trials

In initial analysis for the performance data, the spread of residuals across the fitted values was approximately equal and frequency distribution and QQ plot suggested normally distributed data. However, tests for normality were nevertheless significant. Thus, we used GLMs with a gamma distribution and log-link function to examine the effects of prolonged courtship and morphological characteristics on subsequent locomotor performance [41]. The factor included in the models was treatment (25 ‘control’ or 25 ‘display’ males) and the dependent variable was either ‘distance attained’ or ‘maximum speed’, while ‘body length’ was included as a covariate as body size may influence performance of the locomotion task. Spearman rank correlations were used to test for relationships between performance capacities, male morphology and display vigour.

#### Analysis of mate-choice trials

Here we analyzed data from the 20 males (10 previous ‘control’ and 10 previous ‘display’ males) that engaged in the mate-choice trials. As the proportion of time spent performing full wing-raises was left skewed and could not be corrected, we used a Mann-Whitney U test to test for the effects of courtship vigour and size on mating success. The factor included in the models was mating success (‘successfully mated’ or ‘non-mated’), while the measured variable was either i) the proportion of time spent performing full wing raises, ii) body length or iii) mean wing length. A Wilcoxon signed-rank test was used to test for differences in the proportion of full wing raises performed by the males between the prolonged courtship trials and the mating trials.

## Results

### Courtship and performance capacities

**Fig 2:**
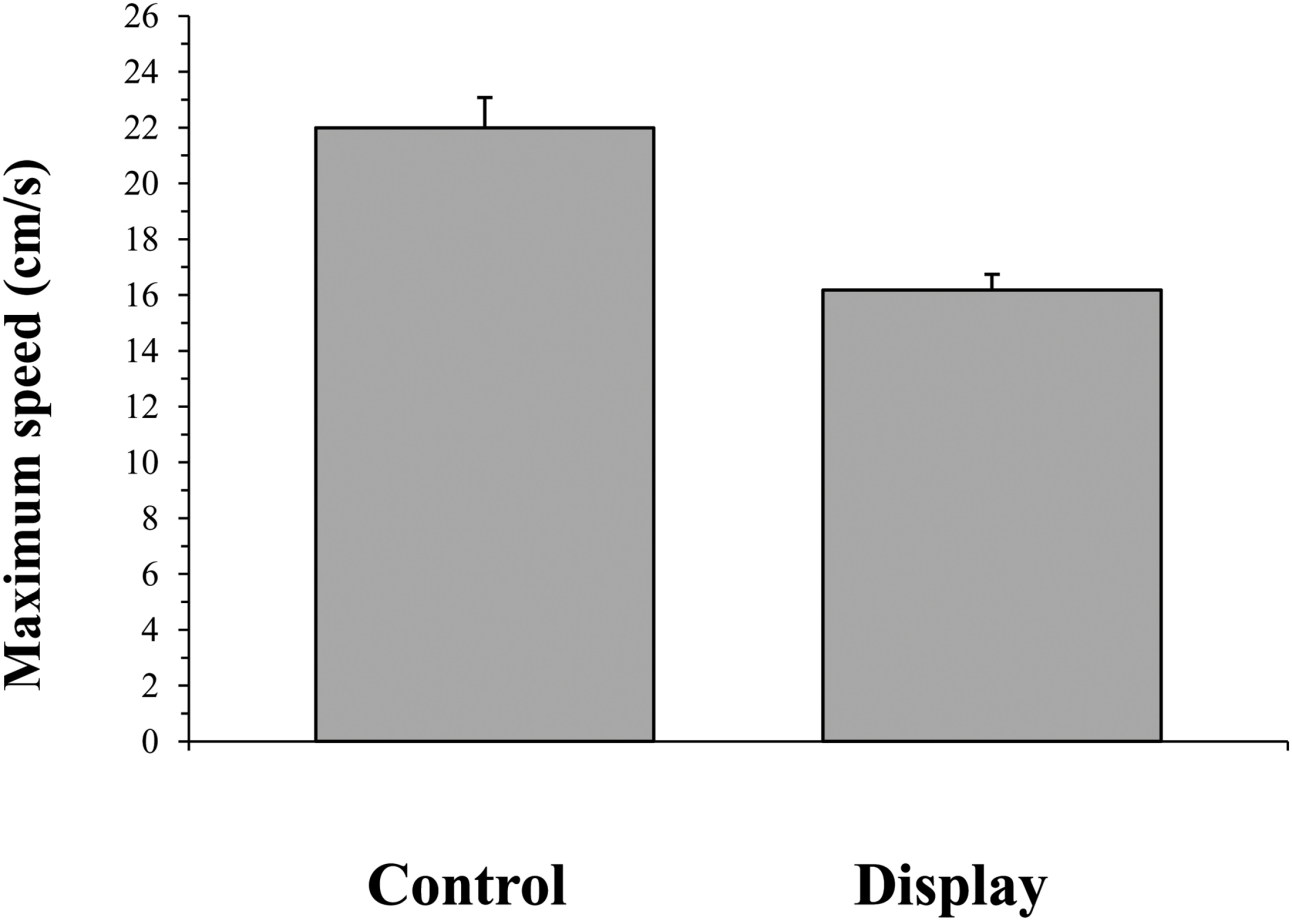
The maximum speed (mean ± SE) attained on the racetrack by control individuals and those that had engaged in courtship display.

**Fig 3:**
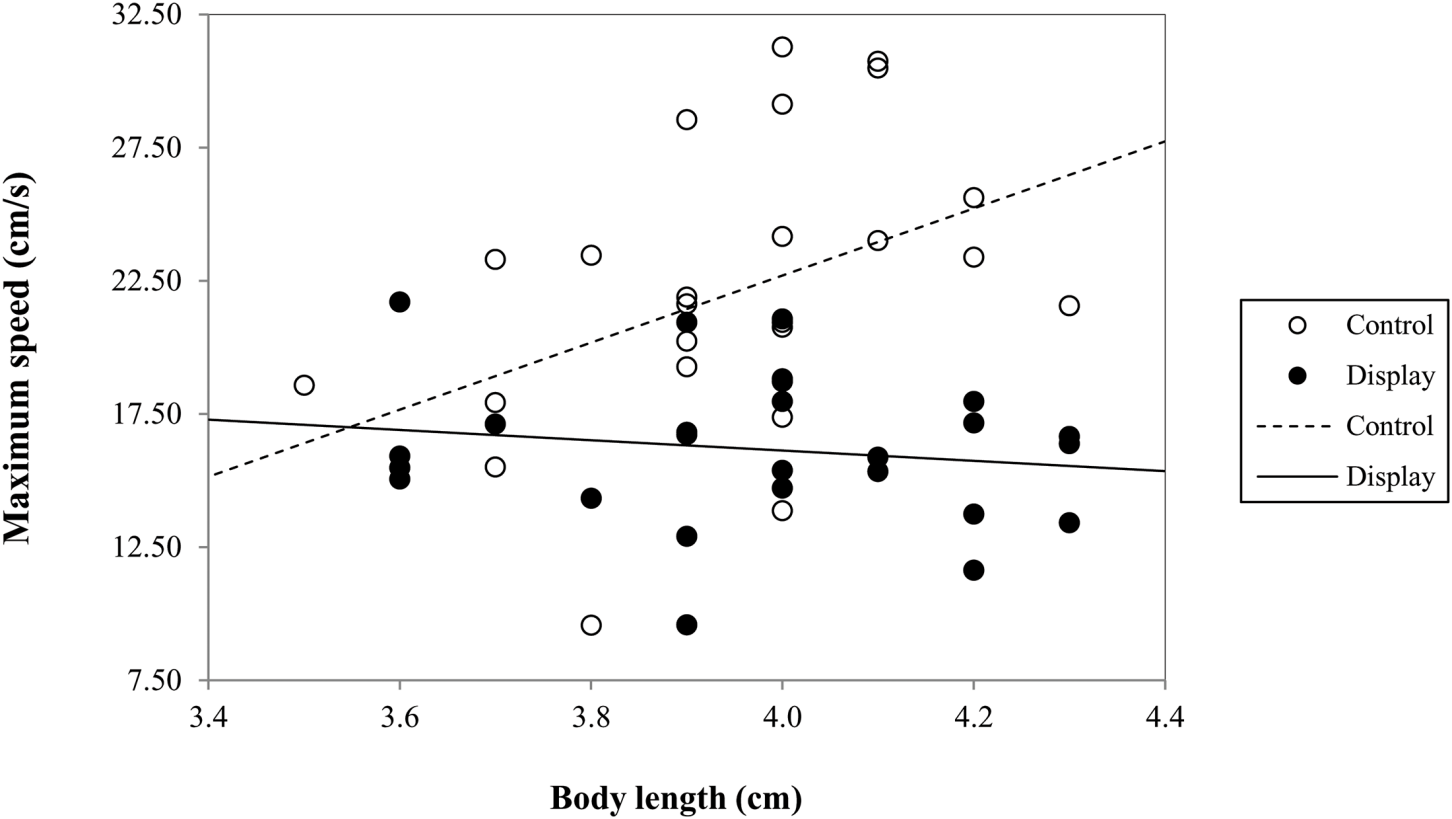
Bivariate scatterplot with fitted regression lines illustrating the interaction between male body length and maximum speed attained by the control individuals (unfilled circles) and males that had completed 360 s of courtship display (filled circles).

Control individuals attained significantly higher maximum speeds in the performance trials than individuals that had engaged in 360 s of courtship display (*F*_1,48_ = 27.693, *P* < 0.0001; Fig 2). There was no overall relationship between the maximum speed and the body length of the male (*F*_1,47_ = 1.054, *P* = 0.3101), while there was a significant interaction between maximum speed, body length and treatment group (*F*_1,46_ = 5.6321, *P* = 0.0219; Fig 3). There was a significant positive correlation between body length and maximum speed in control individuals (*r*_s_ = 0.499, *df* = 23, *P* = 0.0145; Fig 3), whereas in display individuals there was no such relationship (*r*_s_ = -0.084, *df* = 23, *P* = 0.6806; Fig 3).

Control individuals achieved significantly greater distances in the performance capacity trials than the individuals that had engaged in 360 s of courtship display (control mean ± s.e. = 44.06 ± 2.510 m over five minutes, display mean ± s.e. = 32.45 m ± 1.284 over five minutes; *F*_1,48_ = 20.744, *P* < 0.0001). There was no relationship between the distance attained and the body length of the male (*F*_1,47_ = 0.8153, *P* = 0.3713), but there was a significant interaction between distance covered, body length and treatment group (*F*_1,46_ = 4.209, *P* = 0.0459). There was a significant positive correlation between body length and distance covered in control individuals (*r*_s_ = 0.468, *df* = 23, *P* = 0.0218), whereas in display individuals there was no relationship (*r*_s_ = -0.097, *df* = 23, *P* = 0.6363).

### Courtship vigour and performance capacities

There were no significant relationships between maximum speed and the number of full wing raises completed (*r*_s_ = 0.153, *df* = 23, *P* = 0.4544), or the total duration of full wing raises performed (*r*_s_ = 0.100, *df* = 23, *P* = 0.6235). Similarly, there were no significant relationships between distance covered and the number of full wing raises completed (*r*_s_ = 0.219, *df* = 23, *P* = 0.2832), or the total duration of full wing raises performed (*r*_s_ = 0.154, *df* = 23, *P* = 0.4510).

### Male size and display vigour

There was a significant positive correlation between body length and mean wing length (*r*_s_ = 0.805, *df* = 48, *P* < 0.0001). There was no correlation between male body length and the number of full wing raises completed (*r*_s_ = -0.070, *df* = 23, P = 0.7309), or the total duration of full wing raises performed (*r*_s_ = -0.146, *df* = 23, p = 0.4752).

### Mate-choice trials

#### Effects of repeated courtship

In the second set of trials, where females were free to mate with the males, there was a non-significant trend for a lower proportion of full wing raises performed by males that had displayed the previous day compared to the previously non-displaying control group (*U* = 26, *N* = 20, *Z* = -1.814, *P* = 0.0696). A Wilcoxon signed-rank test revealed that males produced a marginally higher proportion of full wing raises during the mating trials (day 2) than during the courtship trials (day 1) (Z = -1.960, *P* = 0.0499)).

#### Female choice and male vigour

**Fig 4:**
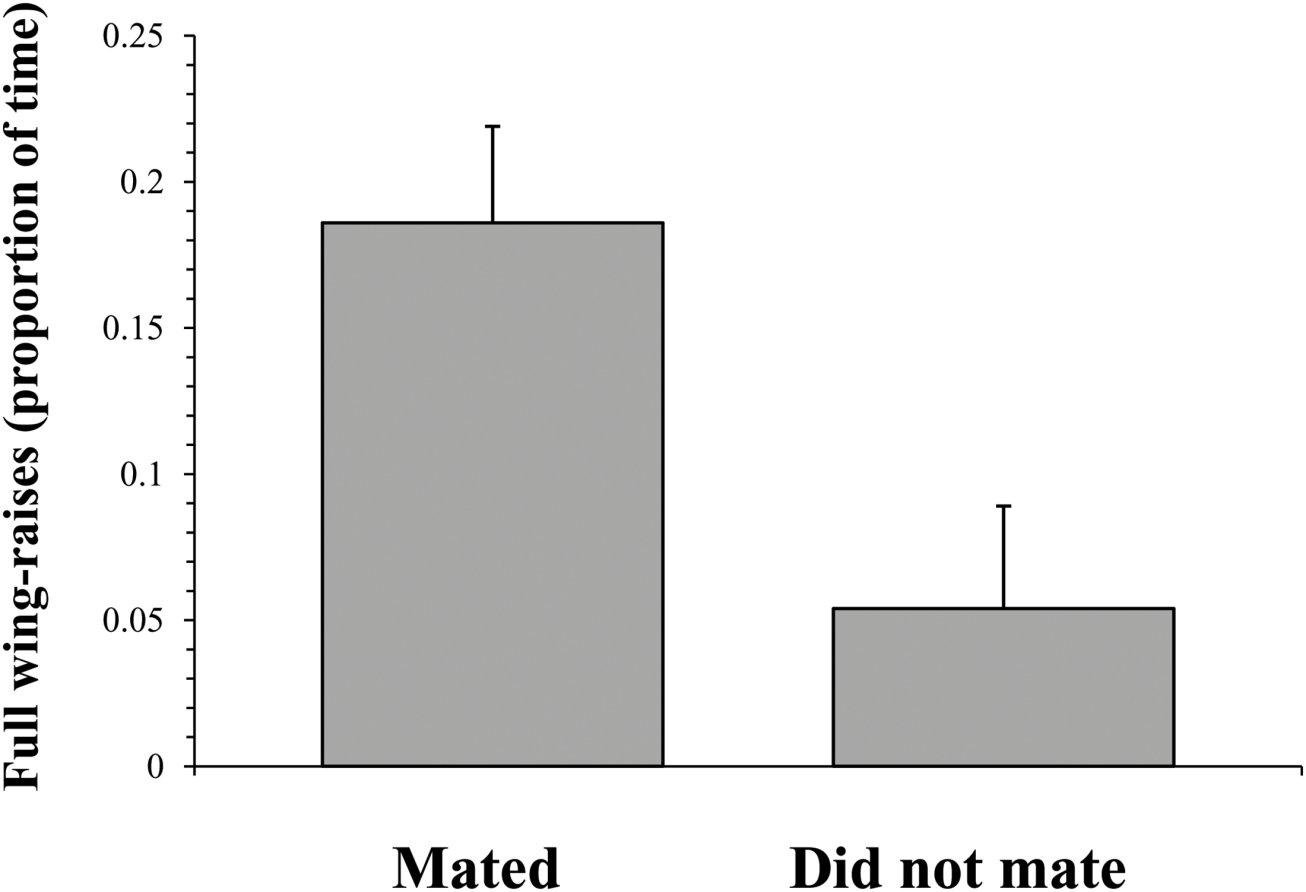
The proportion of full wing-raises (mean ± SE) performed by males that went on to mate with the female and those that did not.

There was a significant difference between the proportion of full wing raises performed by males that were mated with (*N* = 12; previously courted = 5, former control = 7) and those that were not (*N* = 8; previously courted = 5, former control = 3), with successful males performing a greater proportion of full wing-raises during the mating trials than unsuccessful males (*U* = 15, *N* = 20, *Z* = -2.546, *P* = 0.0109; Fig 4).

#### Female choice and male morphology

There was no difference in the body length of males that were mated with and those that were not (*U* = 40.5, *N* = 20, *Z* = -0.579, *P* = 0.5628) or in the mean wing length of those males that were mated with and those that were not (*U* = 38, *N* = 20, *Z* = -0.772, *P* = 0.4404).

## Discussion

A recurring theme in the study of repetitive signals is that the repeated elements of a display may combine to produce an energetically costly signal that advertises the stamina of the sender [6, 7]. In the present study, male *Byrsotria fumigata* that had engaged in courtship display towards a female were found to have significantly reduced performance in locomotor trials relative to non-displaying control individuals. During courtship interactions, male *B*. *fumigata* perform a dynamic courtship display where they repeatedly raise their wings up and down. The repeated presentation of the wings in this manner could serve two distinct functions. Firstly, the wings may have ornamental characteristics and the function of their repeated presentation may be to allow the female further opportunities to assess male quality via a process of signal validation [5, 7, 9]. In the absence of any distinctive ornamental features, we investigated wing size as a static morphological trait that may be advertised via a signal validation process. Wing size would correlate with body size and may thus advertise this characteristic to the female. However, in the mating trials, females did not appear to exhibit a preference for larger males and female preferences were not based on wing size. Thus, the repetitive wing-raising courtship display is not consistent with signal validation.

The second hypothesized function of signal repetition is that of demonstrating the capacity to bear signaling costs. In this scenario, the duration and vigour of the repeated display would demonstrate the ability of the male to bear either extrinsic (i.e. circumstantial) or intrinsic (i.e. energetic) signaling costs [6, 7, 9]. Indeed, we found that males that had performed a prolonged courtship display gave significantly lower performances on the racetrack than non-displaying control individuals. They obtained significantly lower maximum speeds (‘maximum exertion achieved’) and covered significantly lower distances (‘overall rate of performance’) [41], suggesting that future sprint capacities and endurance capacities were both handicapped as a result of display. Further, although the sample size of the mating trials was fairly small, it still appears that males performing wing-raises with greater vigour were more likely to successfully attract a mate than those that displayed less vigorously. These findings are consistent with a display of stamina advertising the intrinsic cost capacity of the displaying male [6, 7, 9]. It is interesting that there was no relationship between the maximum speed attained on the racetrack and the vigour of the wing-raising display produced. A correlation between natural variation in courtship performance and natural variation in running performance is necessary to fully conclude whether males that court more vigorously have higher stamina and thus whether females are assessing this quality while making their mate choice decisions. One possible explanation for this lack of correlation is that during the courtship process, despite variations in male quality, the courting males all perform at the maximum level of which they are capable and thus equally approach their energetic cost thresholds. Thus, consistent with a quality handicap [11], better-quality individuals perform a more vigorous display before this threshold is reached, whereas for poorer-quality individuals this threshold is lower, being reached by the performance of a less vigorous display. It is therefore possible that individuals that had courted all entered the performance trials with the same level of fatigue. Future studies that allow individuals a sufficient rest period post-display before testing performance capacities [41] may be necessary to support this hypothesis. This procedure would resolve any relationships between an individual’s performance capacity and display vigor. Another possible solution would be to take physiological samples of energetic byproducts immediately post-courtship [26] in order to reveal each individual’s energetic investment in the repeated courtship display.

In addition to reduced locomotor performance as a result of courtship, engaging in courtship also had the effect of nullifying a positive relationship between male size and performance capacities, which remained present in control individuals. In performance trials, it would be intuitive that larger individuals would be capable of attaining greater speeds and covering more ground than smaller individuals. It was thus surprising that we found no overall relationship between the body length of the cockroaches and their performance in the locomotor trials. However, there were significant interactions between the performance measures, body length and treatment groups (‘control’ or ‘display’). There was a significant positive relationship between body length and maximum speed in control individuals, whereas display individuals exhibited no such relationship. Thus it appears that larger cockroaches do have greater performance capacities than smaller individuals, but that this relationship is removed once the individuals are already partially fatigued by the performance of the repetitive courtship display. Thus, not only do the males incur costs as a result of producing this courtship display, but it appears that larger males incur disproportionately larger costs compared to smaller males.

Costs and constraints are critical for the evolution of honest signals of quality, but often we have a poor understanding of what these costs and constraints might be [50]. In the present study, male *Byrsotria* appear to be subject to energetic costs as those that have performed a repetitive wing-raising courtship display have reduced locomotor performance. Thus, in theory, only males with higher stamina can afford the costs of a vigorous display, in which case this energetically costly display would be informative for females, and they should prefer males that perform more vigorous displays. The fact that male *B*. *fumigata* are fatigued by a courtship display produced by repetitive wing movements may be significant from an evolutionary perspective. Insect flight muscle is extremely fatigue resistant due to the lack of lactate dehydrogenase (i.e. no lactate build up, see [51, 52]. This is intuitive for insects where flight is a crucial behavior facilitating escape and dispersal as muscle fatigue would be very costly under these circumstances. However, in groups where species have become flightless (e.g. Gryllid crickets), we have seen that males do indeed accumulate lactate as a direct result of wing movements during courtship (e.g. in *Gryllus bimaculatus*; [26]), demonstrating that prolonged wing muscle use can result in anaerobia. Further, in *G*. *firmus*, where males can be of either a flightless or dispersive morph, females prefer the songs from flightless males [53]. This may represent a movement towards costly signaling, driven by female preferences for males that can signal high energetic cost capacities. Thus, where natural selection on dispersal has driven the evolution of metabolically efficient wing musculature in insects that undergo flight, those that have secondarily lost this capability now appear to have reverted to more energetically costly wing movements where used in display.

This is the first time that performance capacity techniques have been used to demonstrate the performance handicaps incurred as a result of producing a dynamic repeated courtship display. By stimulating an animal to undergo strenuous activity, investigators can replicate the animal’s capacity to perform demanding, ecologically relevant tasks such as evading predators or engaging in lengthy pursuits. Previously, performance techniques have revealed correlations between costly signals used in aggression [34, 38, 42], or predator deterrence [39] and the ability to complete demanding performance trials. These correlations demonstrate the honesty of such signals in accurately reflecting the overall condition of the signaler in terms of the capacity that it is advertising, such as bite force or escape velocity in *Anolis* lizards producing anti-predator signals [39], or signals of fighting ability and stamina in shell-rapping hermit crabs engaged in contests over gastropod shells [41, 42]. It is intuitive that such signals are likely to contain information about the stamina of the sender as this should be related to its ability to escape a predator or fight a rival. However, signals of stamina are equally important in a courtship context, because, among other things, the fighting ability and evasion ability of a male should be relevant to a female interested in provisioning her offspring with these qualities, thus ensuring their fitness [18]. Until now, however, such a premise has not been tested. Here we demonstrate that not only is a courtship display associated with the locomotory performance of the sender (used as a proxy for stamina), such that those that have displayed have depleted performance in locomotor trials, but also that females seem to attend to the vigour of these energetically costly displays when making their mate choice decisions. Thus, females appear to be assessing males on their ability to produce a vigorous display in the face of underlying energetic costs.

Here we have shown that a dynamic repeated courtship display is energetically costly enough to handicap future motor performance and that females likely use this display to inform their mate choice decisions. Similar dynamic repeated courtship displays occur across a wide range of taxa and utilize a suite of modalities including repeated acoustic, motion, electric or seismic signals. It is possible that dynamic repeated displays are so widespread precisely because they are inherently costly and therefore reveal a male’s ability to withstand those costs, ensuring signal honesty. The physiological effects of the repetitive signaling behavior may ensure the honesty of the behavioral display, favouring female preference and thus supporting the coevolution of female preference and male display. We suggest that these displays may serve similar functions in a wide range of taxa and urge researchers to consider the function of repetitive courtship displays in their study systems, where they may act as simple signal validation techniques or function as costly signals.

## Supporting Information

S1 File Data used in this study. (XLS)
